# The perceived size of the implicit representation of the dorsum and palm of the hand

**DOI:** 10.1371/journal.pone.0230624

**Published:** 2020-03-23

**Authors:** Sarah D’Amour, Laurence R. Harris

**Affiliations:** Centre for Vision Research, York University, Toronto, Canada; University of Exeter, UNITED KINGDOM

## Abstract

The perception of the body and its parts has traditionally been studied using the conscious body image. Here, we determine the implicit representation of the hand. Participants were sequentially shown two life-size images of either the dorsal or palmar surface of their hand. In one interval either the horizontal or vertical dimension of the image was varied using an adaptive staircase, while the other interval contained the full-size, undistorted image. Participants reported which image most closely matched their hand. The staircase honed in on the distorted image that was equally likely to be judged as matching their own hand as the accurate image. The implicit representation was taken as midway between these two images. The experiment was repeated with different hand orientations. Perceived width depended on the orientation, with differences found between the upright and right orientations. Interestingly, the perceived length of the dorsum and palm were different from each other—length of the dorsum was overestimated whereas palm length was perceived accurately. This study reveals distortions of the implicit representation of the hands in healthy individuals.

The perception of our hand size is critical for our interactions with the world—especially for finer work. The relative lengths of our fingers and the proportions of our hands are part of defining ourselves. The hand is also our primary organ for exploring the world by touch. Although the palmar side of the hand (specifically the finger tips) is the part used for touching things (with its covering of glabrous skin and notorious concentration of tactile sensors), it is the relatively insensitive dorsum of the hand (covered largely with hairy skin) that is generally visible.

The hand has been the main focus for studying various aspects of perception and action such as touch, haptics, motor skills, reaching, grasping, pointing, tool use, position sense, size perception, etc. [1–14] and so it is important to get a complete picture of how the hand is represented in the brain. All these sensory processes and tasks require knowledge about how the brain represents the hand and therefore can be impacted by any size or shape distortions that may occur. We recently developed a novel psychophysical method for determining perceived body size that provides an objective, unbiased, implicit measure of the body representation in the brain [15,16]. Here, we examine the perceived size of the dorsum and palm of the hand to determine and compare baseline size accuracy when the hand is viewed in various orientations and to compare the representation of the two sides of the hand.

Previous studies looking at how the hand is represented have used methods that involve making judgements based on the perceived location of landmarks, such as the knuckles and fingertips [1,2,7–14,17–20]. These judgments require participants to point to landmarks on their occluded hand, from which maps of perceived size are produced by comparing differences between judged and actual position. This type of task is effective at determining a body model that is based on position sense and postural information of the hand but is potentially complicated by inaccuracies in proprioception, reaching, etc. Here we obtain a measure of the representation of hand size that does not rely on judgements of landmarks or other perceptual tasks but rather looks at the hand as a whole and requires making only size judgements.

Previous studies that have explored hand representation usually focused on one side—either the dorsum or the palm [1,20–23]–but have not considered how the size perception of the two sides of the same body part might correspond. While the perceived sizes of the dorsum and palm have not previously been compared within individuals, previous studies have shown differences between the dorsum and palm for tasks involving localization of cutaneous stimuli [23], mental rotation of the hands after experiencing the rubber hand illusion [24], tactile size perception [10,25], tactile distance [26], position sense [11], pointing to locations of hand and finger landmarks—such as knuckles and fingertips [11] and distances between two fingers [27].

Longo and Haggard [11], in what they referred to as a 2.5-D representation of the human hand (inspired by David Marr [28]), found clear overestimation of the perceived size of both sides of the hand but the palmar side was significantly less overestimated than the dorsal surface. This was despite the fact that the palm, with its larger number of receptors has a larger representation in the primary somatosensory cortical map than the dorsum [29]. Higher accuracy for the palm may be due to the more orderly, somatotopic representation of glabrous skin (palm) compared with hairy skin (dorsum) which is represented irregularly in islands of cortex intermixed with the map of glabrous skin [30]. Tactile skin perception and position sense [10] both show that the implicit representation of the palm is less distorted than that of the dorsum; one might expect that the more accurate representation of the palm would be transferred or extruded to the dorsum if the brain used a single, coherent model of the hand as a volumetric object.

Our study has two motivations. Firstly, to determine how accurate healthy populations are at judging the size of the dorsum and palm of their hand. Based on previous research [1,21,22] we predicted that the dorsum would overall be perceived as shorter and wider than it actually was. Secondly, to assess how perception of hand size might be affected when viewing the hand in familiar and unfamiliar perspectives. The hand is more likely to be identified as belonging to the self when viewed in a familiar orientation [31,32]. Our previous rationale for varying the orientation of the face while assessing its perceived size [15] had to do with unique properties that the face lacks compared to much of the rest of the body especially the hands, properties such as interaction, movement, and visual perspective. We used the opposite line of thought in the current study. The hand has all these attributes and uses them to the greatest extent of any body part, thus making it worthwhile to explore how orientation impacts its perceived size. With all the different positions and forms that the hand can take, it seems natural to consider how perception may change with these varying viewpoint. Why might one assume that measuring it in only a “typical” or “canonical” position would be correct? Life-long experiences of directly interacting with and seeing the hands from multiple different viewpoints may result in greater flexibility and plasticity in how the brain represents and perceives the hand. Thus, unlike for the face, we did not expect that varying orientation would affect the hand’s perceived size.

As in our previous papers [15,16], we included sex and body satisfaction as variables to assess how they might impact body size perception. Since the hand is a relatively neutral body part for weight, shape, and size concerns, we did not have any set predictions about differences that might occur but instead used sex and body satisfaction as exploratory factors to add and extend the existing literature.

## Methods

### Participants

40 participants (20 females, mean age 24.43 years, SD = 9.37) took part in the experiment. Handedness was assessed with the Edinburgh Handedness Inventory [33] to ensure that only those with a strong preference were included (38 right-handers). They were recruited from the York University Undergraduate Research Participant Pool and received course credit for taking part in the study. The experiment was approved by the York Ethics Board and all participants signed informed consent forms. The study was performed in accordance with the Treaty of Helsinki.

### Materials/Stimuli

#### Body dissatisfaction

The Body Shape Questionnaire (BSQ) [34] is a 34-item self-report questionnaire that was developed to assess concerns about body shape and experiences of feeling fat that participants may have experienced within the previous month. Each question uses a scale from 1 (never) to 6 (always) that are added together to obtain a total BSQ score ranging from 34–204, with lower scores indicating lower levels of body dissatisfaction and higher scores indicating higher levels of body dissatisfaction. The test was administered before the experiment began to obtain a measure of body dissatisfaction. The total BSQ score was used for correlations. To create categorical groups, participants were divided into high and low groups according to whether their score was above or below the overall mean.

#### Photographs

Color photographs of the dorsal and palmar surfaces of the participant’s dominant hand placed flat against a wall were taken using a digital camera (Canon EOS 10D). Photographs were taken under standardized conditions with the flash on, from midarm to the fingers, from a distance of approximately four feet (122 cm) with no zoom function. The images were then cropped to include only the dorsum or palm and formatted with a white background (Adobe Photoshop CC 2014). These images served as the undistorted “real” images and were used for composing the distorted images. Using a soft tape measure, hand size was measured straight down from the tip of the middle finger to the bottom of the hand, just before the start of the wrist. The size of the image was presented life-size by adjusting the magnification of the image until it matched the participant’s actual hand size. Optical distortions of the lens were confirmed as being less than 0.3% of the width or length of the photograph (given that the other length was set to veridical) using Matlab to assess the amount of distortion.

#### Distorting the images

**Fig 1:**
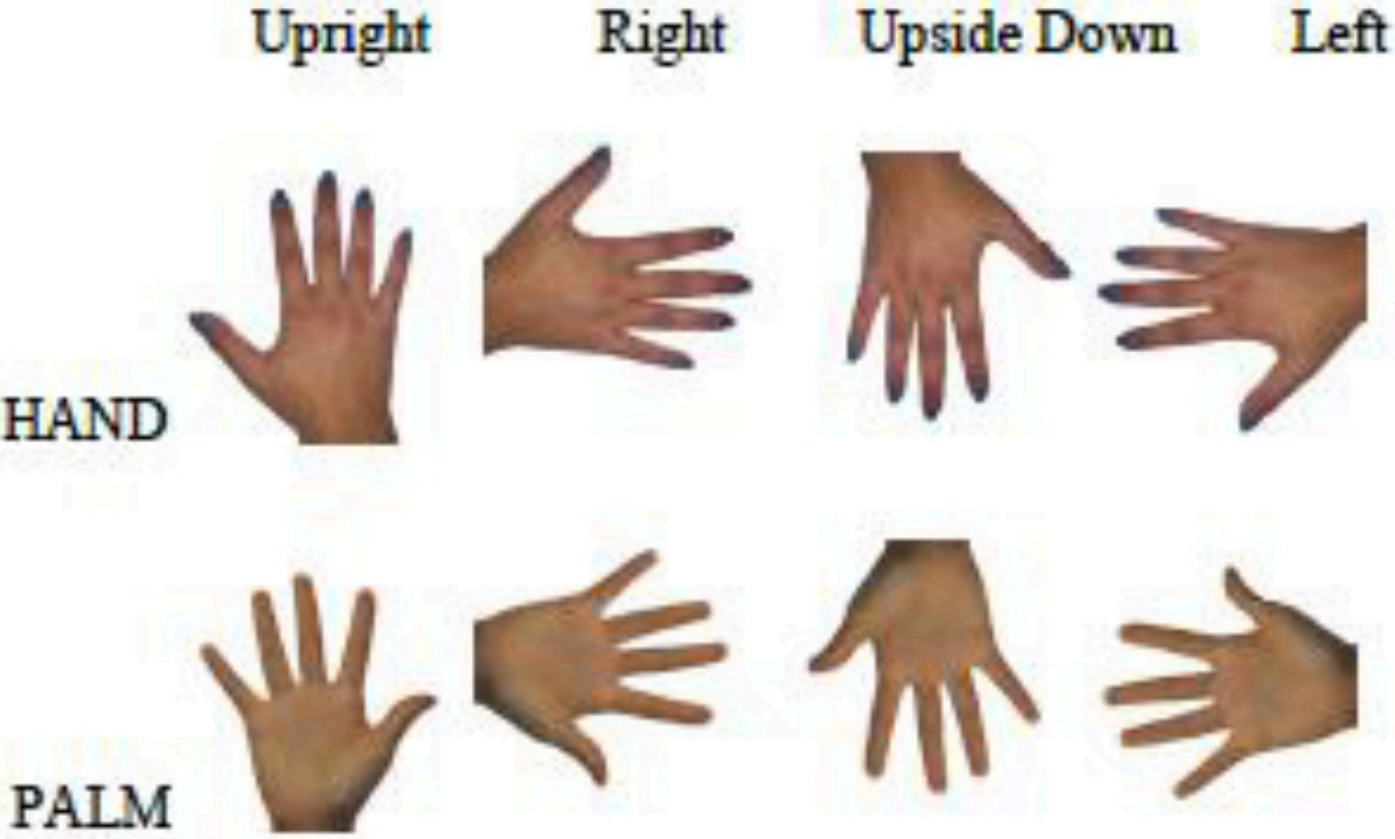
Sample images for the dorsum and palm in each orientation used. The four orientations are shown, from left to right, in the order of (1) upright (0°), (2) to the right (90°), (3) upside down (180°), and (4) to the left (270°) for the dorsum (top row) and palm (bottom row).

Images were presented and distorted using MATLAB (version 2011b) and Psychophysics Toolbox [35] running on a MacBook Pro. One dimension of the image (either width or length) was distorted (made either bigger or smaller) using a QUEST adaptive staircase psychometric procedure [36]. The image was viewed in the center of a computer monitor (27-inch Apple display) with the dorsum or palm in one of the four orientations shown in Fig 1: (1) upright (0°), (2) to the right (90°), (3) upside down (180°), and (4) to the left (270°). Perceived width and length dimensions were separately measured for the palm and dorsum in each of the four orientations, resulting in a total of 16 conditions.

### Procedure

Participants sat in a chair approximately 45 cm in front of the computer screen. To measure perceived size, a two-alternative forced choice (2AFC) design was used. Each trial consisted of two 1.5s intervals—one interval containing the undistorted image and one interval containing the distorted image—separated by a blank white screen for 1.5s. Participants identified which interval contained the image that most closely matched the perception of their own hand and responded using a two-button computer mouse (left button for first interval, right button for second interval). Two interleaved QUEST staircases (25 trials per staircase) were used for each condition (50 trials total), with one starting with the manipulated dimension 40% larger than the undistorted image and the other starting with that dimension 40% smaller than the undistorted image. The standard deviation of the guess was set to 60% as the authors [36] suggest a liberal standard deviation to improve accuracy. Each of the 16 conditions were run in a single block and took approximately six minutes to complete. Block order was determined by a Latin square and was counterbalanced across participants.

### Data analysis

The QUEST program returned an estimate of the distortion at which the participant reported that the distorted image was equally like their perceived body size as the undistorted image. The QUEST algorithm assumes the observer's psychometric function follows a Weibull distribution and adaptively determines the amount of distortion to be presented based on the participant's response to the previous trials. As the experiment goes on, knowledge on the observer's psychometric accumulates. To visualize and confirm the QUEST’s performance, the participant’s decision was plotted against the distortion used for each trial and fitted with a logistic (Eq 1) using the curve fitting toolbox in MATLAB.

$$\text{Decision}=1/\left(1+\text{exp}\left(-\left(\text{x}-{\text{x}}_{\text{o}}\right)/\text{b}\right)\right).$$ (1)

where x_0_ is the actual size, x is the distorted value that was equally likely to be judged as matching the observer’s size, and b is an estimate of the slope of the function. The size of the internal body representation was taken as the point halfway between x and x_0_.

All of the participants’ data for each condition were examined to validate the accuracy and efficiency of the QUEST procedure. The QUEST’s performance was also checked to determine whether reliable estimates were obtained within 50 trials. All data were inspected for outliers. Outliers were defined as points that fell outside ± 3 standard deviations from the mean. Data that failed to meet these criteria were discarded from the analyses (0 participants).

Estimates were converted to percentages relative to the accurate reference photograph. For the two left-hand participants, the data for the right and left orientations were switched to correspond to the correct position. Data analyses were conducted on these values using SPSS 23. Correlations and mixed measures analyses of variances (ANOVAs) were used for statistical analyses, with alpha set at *p* < .05 and post-hoc multiple comparisons were made using Bonferroni corrections.

## Results

Data in only one out of 16 of the conditions were significantly correlated with BSQ score so BSQ groups were not included in the analyses.

### Dorsum and palm size accuracy: Width dimension

**Fig 2:**
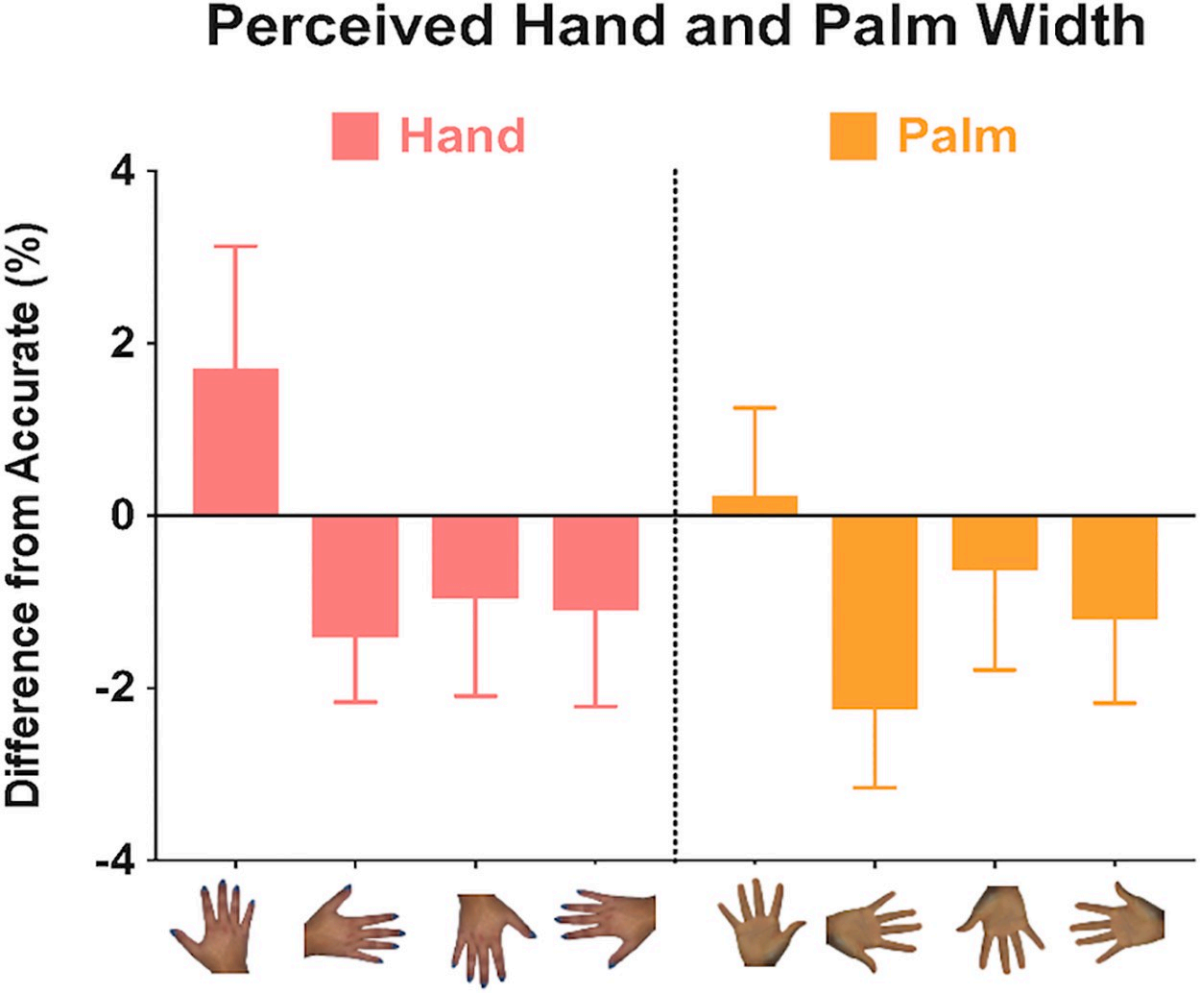
Perceived width of the dorsum and palm. Mean differences from accurate when dorsum of the hand (left panel) or palm (right panel) was distorted in the width dimension for each viewing orientation. Positive and negative scores represent overestimation and underestimation respectively. Error bars represent ±1 SEM.

Fig 2 shows the results for perceived width of the dorsum and palm. A three-way mixed ANOVA was conducted to test for the within-subjects effects of body part (dorsum or palm), orientation (upright, right, upside down, and left), and the between-subjects effect of sex (male and female). The only significant effect found was for orientation, *F*(3, 114) = 3.44, *p* = .018, ηp2 = .083, with pairwise comparisons showing that upright (*M* = .97, *SE* = .95) and right (*M* = -1.82, *SE* = .67) orientations differed by 2.79% ± .98, *p* = .044, 95% *CI* = [.05, 5.53].

### Dorsum and palm size accuracy: Length dimension

**Fig 3:**
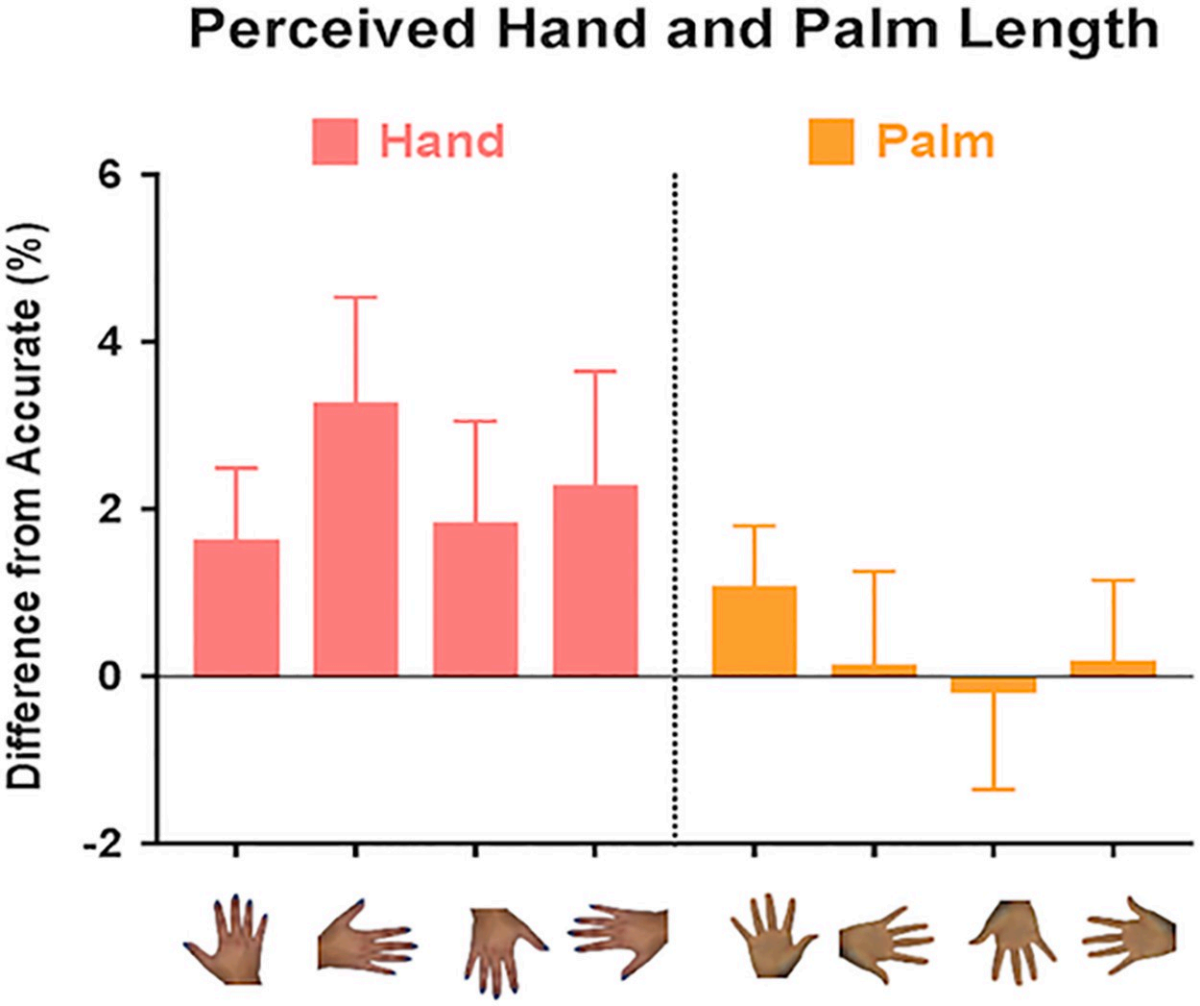
Perceived dorsum and palm length. Mean differences from accurate when dorsum (left panel) or palm (right panel) was distorted in the length dimension for each viewing orientation. Positive and negative scores represent overestimation and underestimation respectively. Error bars represent ±1 SEM.

A second ANOVA was conducted using the same variables as above but for the length dimension (Fig 3). A significant main effect was found for body part, *F*(1, 38) = 8.68, *p* = .005, ηp2 = .186, with greater amounts of distortion observed for the dorsum of the hand (*M* = 2.26, *SE* = .94) compared to the palm (*M* = .30, *SE* = .74). This means that participants were less accurate at judging the length of their dorsum but were not significantly different from perfect accuracy for the palm.

### Relationship between dorsum and palm

**Fig 4:**
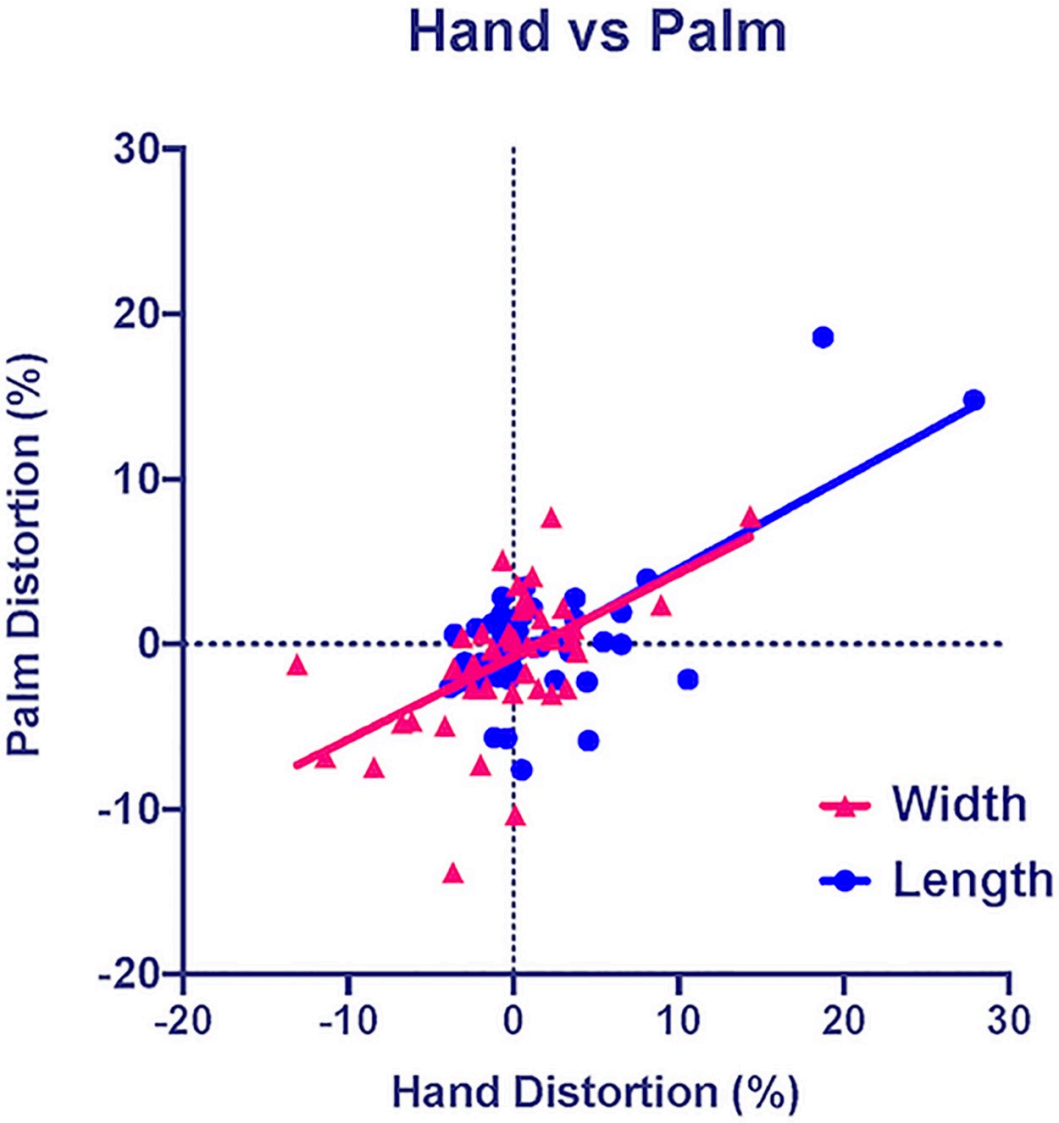
Dorsum vs palm. Relationship between dorsum and palm distortions for width (pink triangles) and length (blue circles). The lines plotted through the data are the goodness-of-fit lines for the width and length.

Correlations between errors in the perceived size of the dorsum and palm (Table 1) were conducted for width and length (averaged across the four orientations in Fig 4). The width of the dorsum and palm were significantly correlated, *r*(39) = .550, 95% *CI* = [.29, .74], *R*^*2*^ = .302, *p* < .001, as were the length of the dorsum and palm, *r*(39) = .708, 95% *CI* = [.51, .84], *R*^*2*^ = .502, *p* < .001. These results show that there was a strong relationship between the perceived size of the dorsum and palm–if someone overestimates their dorsum size, they will likely overestimate their palm size as well.

**Table 1:** Correlations between the perceived size of the dorsum and palm (N = 40).

Dorsum vs Palm		Pearson *r*	*p*	*95% CI*
Width	Combined	.550	.000 **	[.29, .74]
	Upright	.157	.334	[-.16, .45]
	Right	.234	.146	[-.08, .51]
	Upside Down	.298	.062	[-.02, .56]
	Left	.243	.131	[-.08, .52]
Length	Combined	.708	.000 **	[.51, .84]
	Upright	.284	.076	[-.03, .55]
	Right	.304	.057	[-.01, .56]
	Upside Down	.365	.021 *	[.06, .61]
	Left	.505	.001 **	[.23, .71]

*p < .05.

** *p* < .01.

### Actual vs perceived size

**Fig 5:**
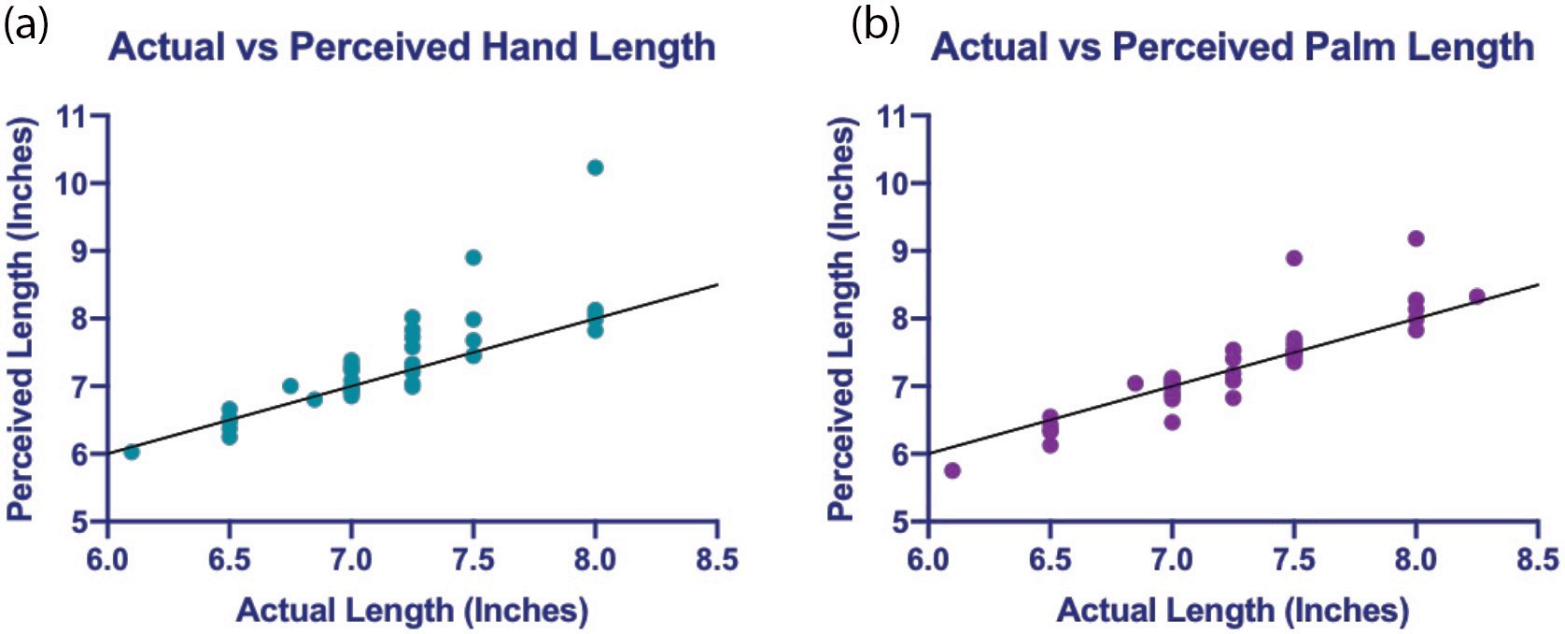
Actual vs perceived length. Relationship between actual and perceived lengths for (A) dorsum length and (B) palm length. The line plotted through the data represents accurate perception.

To see how the distortion percentages would look when calculated relative to actual size, we calculated the perceived lengths of the dorsum and palm (averaged across the four orientations) to make our results more clearly understandable and relatable to previous studies. Fig 5 shows the perceived length of (Fig 5A) the dorsum (*M* = 7.31 inches, *SE* = .12) and (Fig 5B) palm (*M* = 7.27 inches, *SE* = .11) plotted as a function of actual length (dorsum: *M* = 7.14 inches, *SE* = .07; palm: *M* = 7.24 inches, *SE* = .08). The perceived length was significantly difference from the actual length for the dorsum, *t*(39) = 2.37, *p* = .023, ηp2 = .125, with people perceiving their dorsum to be longer than it actually was by .17 inches ± .07, 95% *CI* = [.02, .32]. There was no significant difference between the perceived and actual length of the palm, *t*(39) = .57, *p* = .573, ηp2 = .008.

## Discussion

The method we used here reveals distortions of the implicit body representation independent of the conscious body image. The length of the representation of the dorsum and palm of the hand were significantly different from each other but only the perceived width of the dorsum depended on orientation with overestimation found when the dorsum was viewed upright and an underestimation when viewed in the right orientation. The representations of the dorsum was longer than its actual size. The dorsum and palm did not show any differences for width judgements. Accuracy differed between the dorsum and the palm in the length dimension, meaning that the perceived length of the dorsum and palm were not the same although there was a strong correlation between them.

Perception of the size of the dorsum and palm reveals distortions in how the body is represented in the brain. The enormous distortions in the homunculus of the primary somatosensory cortex are largely corrected [37] but for every orientation, dorsum length was overestimated whereas the length of the palm was perceived accurately. This is curious as it is the dorsum that is more usually seen (look down at your hands as they type on your computer right now). However, this makes sense in terms of function, as it is the location of the fine-tuned receptors on the palm side of your fingertips [38] that it is important that you place in the correct positions.

### Comparisons to previous findings

Overestimations of hand width, underestimation of finger length, and a radial-ulnar gradient of magnification, with finger length underestimation increasing from thumb to little finger were found by Longo & Haggard [1]. Longo & Haggard [10] confirmed these findings and also showed that the width of both the dorsal and palmar surfaces were overestimated whereas only finger length was underestimated for the dorsal surface. These findings differ from our current findings but there are many explanations for why this might be– 1) our methods are extremely different including the design, task, and measures obtained; 2) they measured hand width using the distance between the bases of the index and little fingers; 3) they only looked at finger length rather than the length of the entire hand and palm. In fact, no distortions were found when they tried using a template matching task to see how it would compare to their original findings [1,12,39].

### Sex and body satisfaction scores

Since sex [40–43] and body satisfaction [44–47] are known to be factors that influence body perception, we looked at both variables in our current study but surprisingly did not find effects of either.

Our findings that there are no differences between how males and females perceive their hand size is not compatible with what has been found in previous studies that looked at sex differences in hand representation [6,20]. Longo [6] recently conducted a meta-analysis of nineteen experiments that used his ‘psychomorphometric paradigm’ method of mapping the body [1] to examine whether sex differences exist for perceptual hand maps. He concluded that distortions occurred for both males and females but that sex differences exist in the magnitude of distortions in perceptual hand maps, with women showing greater overestimation of hand width and men showing greater underestimation of finger length. This differs from what Coelho & Gonzalez [20] found. They found that finger length was underestimated in both sexes but hand width was only overestimated in women. Longo [6] speculates that these differences could be explained by how actual hand size (used as a reference) was measured—using a photograph of the hand next to a ruler in his studies compared to Coelho and Gonzalez’s method of pointing to landmarks on a non-occluded hand. The latter may not provide an accurate reference due to potential motor control or visual biases with which such localization tasks may be associated. Again, the fundamental differences between our experimental methods and how the hand was measured could be the cause (e.g., hand width and finger length) since our method asks people to access their internal representation.

## Conclusions

Knowledge about body size is important for many aspects of perception and action that rely on this information for executing and interpreting tasks, especially for the hands that are required for so many fundamental behaviours (reaching, grasping, touching, holding, etc.,). Distortions in how the brain represents the hand may impact these processes and result in errors that have far-reaching consequences.

## Supporting information

S1 Data (XLSX)
